# Proof of Concept of Integrated Load Measurement in 3D Printed Structures

**DOI:** 10.3390/s17020328

**Published:** 2017-02-09

**Authors:** Michaël Hinderdael, Zoé Jardon, Margot Lison, Dieter De Baere, Wim Devesse, Maria Strantza, Patrick Guillaume

**Affiliations:** 1 Department of Mechanical Engineering, Vrije Universiteit Brussel, Pleinlaan 2, 1050 Elsene, Belgium; zoe.jardon@vub.ac.be (Z.J.); margot.lison@vub.ac.be (M.L.); dieter.de.baere@vub.ac.be (D.D.B.); wim.devesse@vub.ac.be (W.D.); patrick.guillaume@vub.ac.be (P.G.); 2 Vrije Universiteit Brussel, Department of Mechanics of Materials and Constructions, Pleinlaan 2, 1050 Elsene, Belgium; maria.strantza@vub.ac.be

**Keywords:** strain, additive manufacturing, 3D printing, smart structures, tensile, embedded capillary

## Abstract

Currently, research on structural health monitoring systems is focused on direct integration of the system into a component or structure. The latter results in a so-called smart structure. One example of a smart structure is a component with integrated strain sensing for continuous load monitoring. Additive manufacturing, or 3D printing, now also enables such integration of functions inside components. As a proof-of-concept, the Fused Deposition Modeling (FDM) technique was used to integrate a strain sensing element inside polymer (ABS) tensile test samples. The strain sensing element consisted of a closed capillary filled with a fluid and connected to an externally mounted pressure sensor. The volumetric deformation of the integrated capillary resulted in pressure changes in the fluid. The obtained pressure measurements during tensile testing are reported in this paper and compared to state-of-the-art extensometer measurements. The sensitivity of the 3D printed pressure-based strain sensor is primarily a function of the compressibility of the capillary fluid. Air- and watertightness are of critical importance for the proper functioning of the 3D printed pressure-based strain sensor. Therefore, the best after-treatment procedure was selected on basis of a comparative analysis. The obtained pressure measurements are linear with respect to the extensometer readings, and the uncertainty on the strain measurement of a capillary filled with water (incompressible fluid) is ±3.1 µstrain, which is approximately three times less sensitive than conventional strain gauges (±1 µstrain), but 32 times more sensitive than the same sensor based on air (compressible fluid) (±101 µstrain).

## 1. Introduction

Strain measurements are used in multiple steps of engineering projects. Material characterization, design validation and load monitoring of structures are just some examples. To date, most strain sensors (e.g., strain gauges, extensometers, and Fibre Bragg Gratings (FBG)) are externally installed, which often limits their range of use. For material characterization, a test sample can be designed to be compatible with most of the available strain measurement techniques. However, when it comes to design validation, design freedom cannot be addressed via a validation technique, and strain measurements have to be carried out at a given location on the component, with the corresponding shape and size.

The use of strain sensors becomes even more complicated when continuous load monitoring is involved—a new and evolving monitoring technique that forms the basis of condition-based maintenance. In-situ load monitoring allows the extraction of usage information of structures, such as counting fatigue cycles, determining whether loads and usage are according to the design, and ensure load limits are not exceeded during operation. This information can then be used to estimate the remaining operational lifetime and organize maintenance inspections. The monitoring system must remain operational in changing and possibly hazardous environments with limited (visible) access. Environmental influences, drift over time, disbonding, aggressive fluids and other factors can cause early failure of the monitoring system [1,2]. For long-term monitoring, the sensors must be robust and give reliable results for a long time. Using glued sensors, like strain gauges, might not be a good solution, since the adhesive can be affected by humidity, temperature, UV light etc. [3]. A robust, integrated and preferably lightweight monitoring system can overcome these issues.

One can overcome most of the difficulties of long-term load monitoring by embedding a strain sensor into the component, thereby enabling so-called ‘self-sensing’ components [4,5,6,7,8]. So far, most embedded strain measurements were found in matrix materials such as composites and concrete [9,10,11,12,13]. The sensors are embedded in the matrix and cured in afterwards. Additive manufacturing, or 3D printing, now enables such integration of functions inside non-matrix structures such as polymers, metals, ceramics, etc. The layer-wise manner of production allows the inclusion of functionalities inside the component that were not possible by conventional subtractive production techniques.

On the one hand, much research is ongoing to integrate electrical conductors inside polymers to generate built-in sensors. Liquid metal is being inserted in 3D printed microchannels [14,15,16]. Strain gauges have been printed in conductive ink on top of a structure [17] and inside highly stretchable elastomers [18]. Despite their successful operation, many drawbacks have been reported on the use of conductive ink. The curing must happen at an elevated temperature and for a significant amount of time, which might be incompatible with the polymers commonly used in 3D printing. The process is also too costly for large scale production. The conductive ink is approximately 100 times more expensive than ordinary copper, but its conductivity is commonly 30 times less. Therefore, researchers have begun to explore the use of solid metal conductors, such as copper wires, in 3D printing [17]. On the other hand, fiber-optic strain sensors are being embedded in metallic components by means of powder-bed based additive manufacturing [19] and shape deposition manufacturing [20,21].

The current work describes another method of integrating a strain sensitive capillary feature inside 3D printed components, which is not restricted to one material type only. The embedded capillary is filled with a fluid and closed by an externally mounted pressure sensor. The elongation (contraction) of the integrated capillary results in a pressure decrease (increase) of the fluid inside the capillary, which is measured by the pressure sensor. As the pressure sensor can be placed at a convenient location, this method of strain sensing is inherently robust and lightweight. The installation cost of the presented system, for structural health monitoring applications, is limited to the installation of an off-the-shelf pressure sensor. The capillary must be incorporated in a strain affected zone but does not induce any additional manufacturing or installation cost.

**Figure 1:**
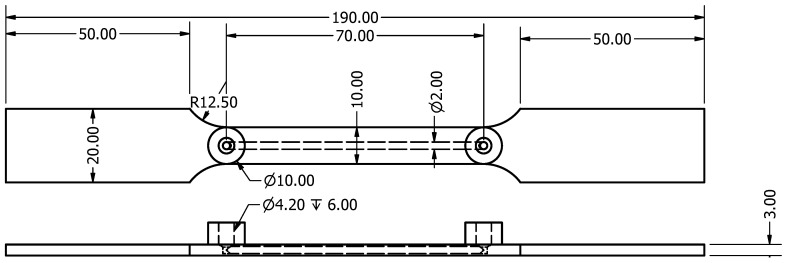
Tensile test sample dimensions (in mm).

In this paper, as a proof-of-concept for the presented strain sensing approach, the capillaries were embedded in tensile test samples (see Figure 1) and the pressure measurements compared to state-of-the-art extensometer measurements. The capillaries were filled with air (compressible fluid) and water (incompressible fluid) to investigate the effect of compressibility of the fluid on the sensitivity of the strain sensor.

## 2. Design

The tensile test samples were designed in order to compare the state-of-the-art extensometer results with the strain measurements of the proposed monitoring system. To that end, the tensile test samples were designed to accommodate both measurement techniques, in order to investigate the concept of load monitoring using integrated capillaries in 3D printed components.

**Figure 2:**
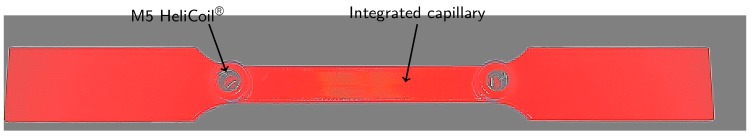
3D printed tensile test sample.

The proposed strain monitoring system consists of an externally mounted pressure sensor connected to a 2 mm diameter capillary integrated into the test section of the tensile test sample. The extensometer measured the strain in the same test section as the integrated capillary. Alongside this first connection, an additional external connection was added to ease the filling of the capillary, flush the channel, and check that the capillary was not clogged. These connections were threaded using M5 HeliCoil’s^®^. Figure 1 gives an overview of the dimensions (in mm) of the tensile test samples and integrated monitoring system. Figure 2 shows a tensile test sample with the HeliCoil^®^ threaded connections installed.

## 3. Materials and Production Process

The tensile test sample were produced by an Ultimaker 2+ (Ultimaker, Cambridge, MA, USA), a 3D printer using Fused Deposition Modeling (FDM) technology. FDM, one of the common variants of additive manufacturing techniques, has been widely adopted within the industry for producing complex geometrical parts at a reasonably low cost [22]. The raw material used is in the form of filament that is melted when extruded through a heated nozzle. The material is deposited while the nozzle is moving in a horizontal plane and the material solidifies at the deposited location. The build plate is lowered when the layer is finished and new material added on top of the previous layer until the component is fully completed.

The embedded capillary is filled with air or water and becomes pressurized when deformed during tests. The embedded capillary must therefore be air- and watertight, which largely depends on the choice of material and production process used. Acrylonitrile butadiene styrene (ABS) is a typical material used for the FDM process and can be made water-impermeable. As printed, the ABS material deposited in layers or as strands is incapable of holding water. In particular, for a (micro) fluidic design with imprinted channels and network geometries this issue is critical. The layers do not blend together to create a watertight device, with small gaps and holes in between the deposited material [23]. It is therefore of interest to chemically modify the surface of ABS to become water impermeable.

Water tightness of FDM printed ABS parts is generally ensured by an after-treatment process involving dimethyl ketone (acetone). Immersion or direct contact with acetone may lead to very quick reaction and may damage ABS parts if the dipping time and concentration are not monitored/optimized accurately. Even a short exposure of ABS to hot vapor may lead to non-uniform treated surfaces [24]. Cold vapor treatment was therefore used in the work of Garg et al. [24]. Other works report the use of acetone-water solutions to treat the ABS surfaces [23,25].

**Figure 3:**
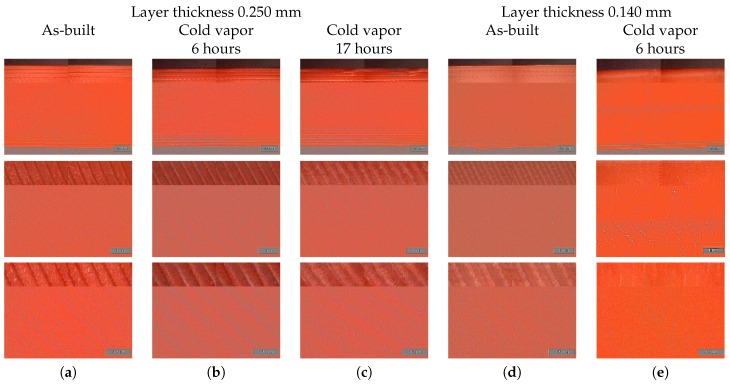
Optical microscope images of external surface of acrylonitrile butadiene styrene (ABS) printed tensile test samples for different acetone-based after-treatment processes and layer thickness.

**Figure 4:**
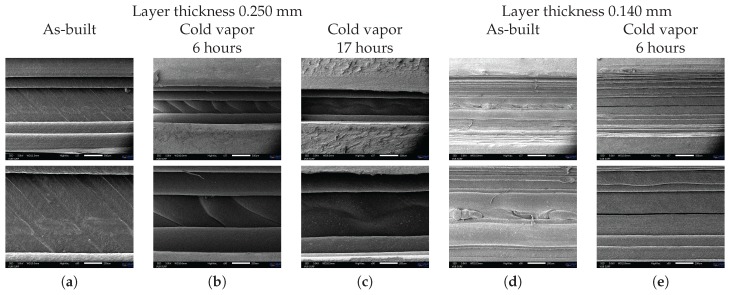
Scanning Electron Microscope (SEM) images of capillary surface inside ABS printed tensile test samples with layer thickness of 0.250 mm for (**a**) as built condition (**b**) cold vapor treatment during 6 h (**c**) cold vapor treatment during 17 h and with layer thickness of 0.140 mm for (**d**) as-built condition and (**e**) cold vapor treatment during 6 h.

In this paper, acetone cold vapor treatment was used in order to melt together the different ABS layers to become water impermeable. A thin layer (5 mm) of liquid acetone was poured on the bottom of a closed container. The samples were held 5 mm above the liquid acetone surface and were turned over every 1.5 h. In order to select a proper duration for the vapor treatment process, the surface roughness and strain sensor performance were compared for as-built, 6 h and 17 h vapor treated tensile test samples. These samples were printed using a layer thickness of 0.250 mm. The optical microscope images, shown in Figure 3a–c, show the bottom side of the tensile test samples (side build plate) in as-built condition, and those that were externally exposed to cold acetone vapor for 6 h and 17 h. It is clear that the cold vapor treatment has modified the surface roughness by blending together adjacent layers. Figure 4a–c shows Scanning Electron Microscope (SEM) images of the inner surface of the capillary inside the tensile test samples with layer thickness of 0.250 mm for different after-treatment processes.

**Figure 5:**
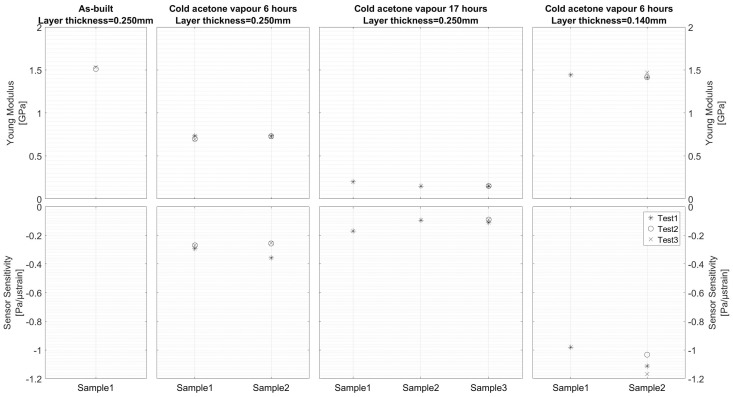
Comparison of cold acetone vapor after-treatment processes on Young Modulus (**top**) and strain measurement system performance (**bottom**) of ABS printed parts.

The strain sensor performance for the different after-treatment processes was evaluated by performing a tensile test and evaluating the sensitivity of the fluid (water) pressure per strain unit. The testing procedure will be discussed in detail in Section 4. The test results are depicted in Figure 5 (bottom). The after-treatment processes also had a major influence on the Young Modulus of the ABS material, as depicted in Figure 5 (top). The first three columns of Figure 5 show a comparison between the as-built condition of ABS material with a layer thickness of 0.250 mm, and after a 6 h and 17 h cold acetone vapor treatment. It is clear from these results that, although a proper acetone treatment is required to obtain a sealed capillary for the strain measurements, long exposure to cold acetone vapor led to a drastic reduction of the Young Modulus and, as a consequence, poor performance of the strain measurement sensor. It was observed that the capillary walls were sucked inwards, which can be seen as the dark sinusoidal irregularities in the capillary wall depicted in Figure 4c. The capillary walls could not withstand the internal vacuum pressure levels, as measured with the samples that were treated for 6 h.

Although the surface roughness was greatly improved by long exposure to cold acetone vapor, the bulk material properties of ABS were greatly deteriorated. It was concluded that the duration of the after-treatment process needed to be reduced, such that only the component’s surface is affected. In order to improve the surface roughness and better squeeze together different layers, a new batch of samples was printed with a reduced layer thickness of 0.140 mm. This second batch of tensile test samples was externally exposed to cold acetone vapor for a period of 6 h, after which the inner capillary surface was treated by sucking cold acetone vapor through the capillary. The acetone vapor was present in the capillary for 2 h, after which it was flushed again with acetone. The flushing procedure was repeated four times. It can be concluded from Figure 3d,e that the surface roughness was indeed ameliorated by the reduced layer thickness. The shape of the capillary was also improved by reducing the layer thickness, as depicted in Figure 4. From Figure 5 it can be seen that the Young Modulus was less affected by the after treatment procedure and the pressure level inside the integrated capillary became very sensitive to strain.

**Figure 6:**
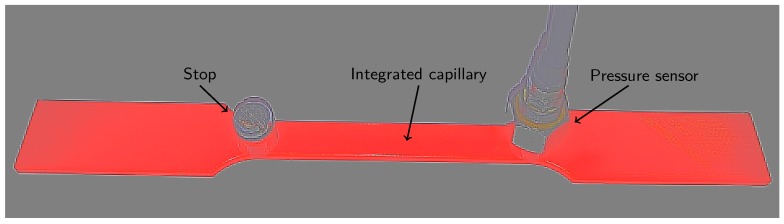
The presented load monitoring system for 3D printed structures.

The latter, improved batch of tensile test samples, was further tested in order to explore the possibilities of the newly developed strain monitoring technique. Two test cases were considered: the capillary of the load monitoring system filled with either a compressible fluid (air) or an incompressible fluid (water). For both cases, the pressure sensor was first installed at one side of the capillary. When the capillary was filled with air, the other end was closed without any further treatment. Only when the capillary was entirely filled with water, was the sample left open at the other capillary end and fully immersed in a bottle of water (with the open connection at the top) and placed in a vacuum oven. This process ensured that all air bubbles escaped from the capillary before closing it at the other end. The fully prepared load monitoring system is shown in Figure 6.

## 4. Testing

The load monitoring system consisted of an integrated capillary filled with a fluid and connected to an externally mounted pressure sensor. The chosen absolute pressure sensor (Kulite XTL-123A-190, Kulite Semiconductor Products, Inc., Leonia, NJ, USA) with a limited range from 0–1.7 bar, is compatible with all nonconductive and noncorrosive liquids or gases.

**Figure 7:**
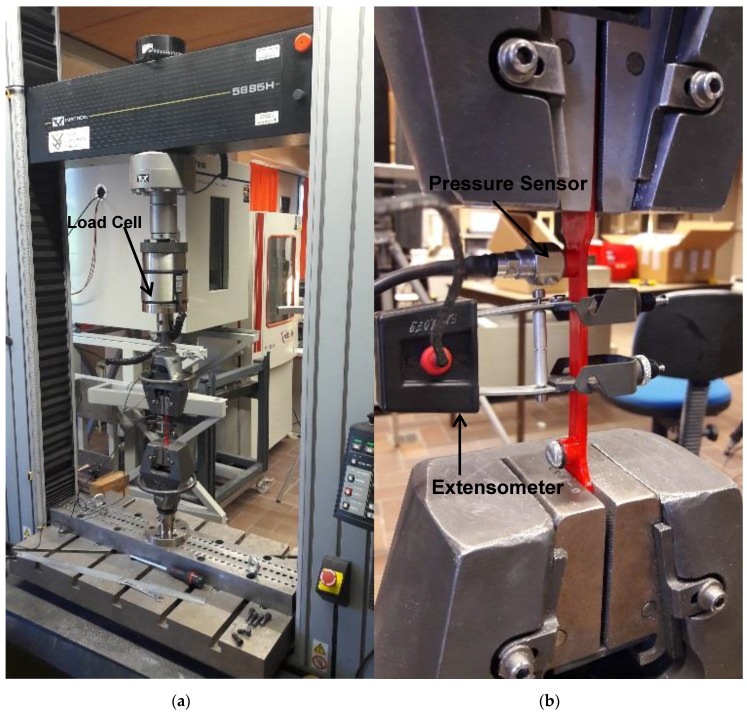
(**a**) Tensile test setup (**b**) Detailed view of the two strain measurements: extensometer and pressure sensor connected to integrated capillary.

The tensile test samples were placed in an Instron 5885 H (Instron, Norwood, CA, USA) tensile testing machine with Instron 2518-103 load cell (10 kN static). An Instron 2630-030 extensometer was furthermore installed on the test section. The pressure sensor, extensometer, load cell and cross head displacement were all recorded using an LMS Scadas III mobile data acquisition system (Siemens, Munich, Germany). All channels were synchronously sampled at a rate of 6.4 kHz. The applied loads were limited to induce low strain levels comparable to the typical elongation of Ti6Al4V at yield:

(1) $$\varepsilon_{Y}=\frac{\sigma_{Y}}{E}=\frac{880\;\mathrm{MPa}}{113.8\;\mathrm{GPa}}=7700\;\mu \mathrm{strain}$$

where $\varepsilon_{Y}$ is the elongation at yield, $\sigma_{Y}$ is the stress at yield and *E* is the Young Modulus of Ti6Al4V. Figure 7 shows the entire setup.

## 5. Results

The sensitivity of the proposed load monitoring system with a capillary embedded within the 3D printed structure is shown in this section, by comparing the pressure measurements with the strain measurements of the extensometer. Subsequently, both the results from the capillary filled with air and water are described, before being compared to each other.

### 5.1. Capillary Filled with Air

**Figure 8:**
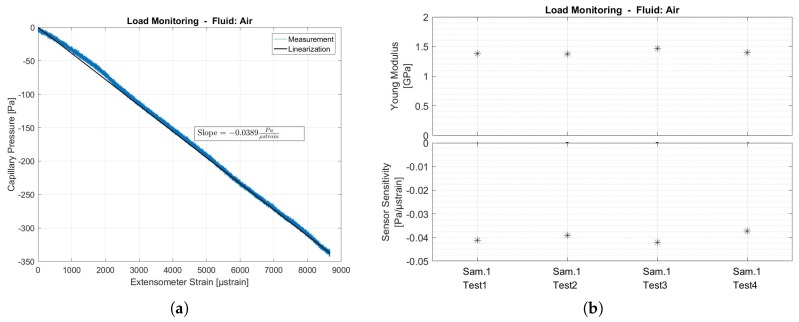
Tensile testing results: Load monitoring system with capillary filled with air is compared to extensometer strain measurements. (**a**) Capillary pressure is linear with respect to extensometer strain (**b**) Comparison of Young Modulus and strain sensor sensitivity of four different tests on same tensile test sample. Raw data can be found in Appendix Figure A1.

The load monitoring system was first tested using a compressible fluid (air) inside the capillary. When under tension, the capillary is elongated and narrowed due to Poisson effects. The net result of the volumetric change of the capillary is positive and the pressure dropped with tension load. Because of the compressibility of the gas, only limited pressure drops were expected. Figure 8 shows all measurement results for the case of compressible fluid in the capillary.

The pressure inside the capillary is proportional to the strain measured by the extensometer on the outside of the same test section. The pressure dropped with a slope of −0.0386 Pa/µstrain The standard deviation of the noise on the pressure was found to be σ=1.3 Pa. Given this standard deviation, 99.73% of all errors due to noise remain within a band of 3σ=3.9 Pa, which leads to an uncertainty of ±101 µstrain.

One way to improve the sensitivity is to install a more precise pressure sensor. However, the accuracy of the load measurement method is mainly limited by the compressibility of the gas inside the capillary. Incompressible fluids are much more sensitive to volume variations and therefore form a better basis for the proposed strain measurement system using embedded capillaries.

### 5.2. Capillary Filled with Water

**Figure 9:**
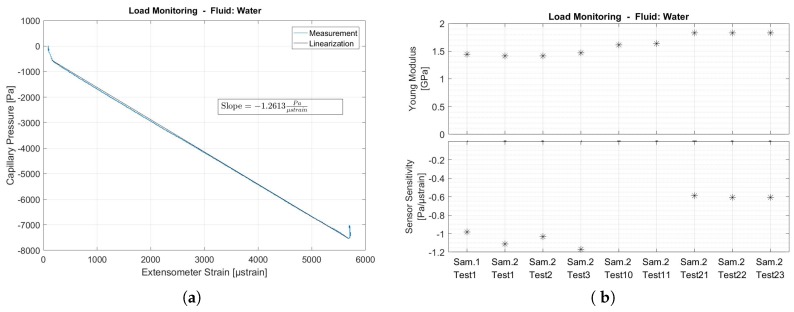
Tensile testing results: Load monitoring system with capillary filled with water is compared to extensometer strain measurements. (**a**) Capillary pressure is linear with respect to extensometer strain (**b**) Comparison of Young Modulus and strain sensor sensitivity of two samples and repeated testing on one tensile test sample. Raw data can be found in Appendix Figure A2.

The same tests as above were repeated for samples with integrated capillaries filled with water. Starting with water at atmospheric pressure in the closed capillary, any deformation of the volume was expected to immediately give rise to a very high pressure change due to the incompressibility of the liquid (water). Therefore, the system signal-to-noise ratio was expected to increase, as for the same noise level (same pressure sensor) a larger signal was expected compared to the previous case. Figure 9 details all measurements of the tensile test and the load monitoring system based on water.

Figure 9a shows a linear relationship between the strain measured by the extensometer and the pressure variations measured by the load monitoring system, except for both ends of the tests where non-linear behavior was observed. As soon as the crosshead started moving, the pressure dropped by approximately 500 Pa, which was recovered when the crosshead stopped moving. In between, the curve fits very well to a linearized curve with slope −1.2613 Pa/µstrain, which is about 32 times more sensitive than the case where the capillary is filled with air.

The water-based load monitoring system was much more sensitive to strain variations than the air-based load monitoring system. Given the standard deviation of the noise on the pressure sensor (*σ* = 1.3 Pa), 99.73% of all errors due to noise remain within a band of 3σ = 3.9 Pa, which now gives an equivalent uncertainty of ±3.1 µstrain on the strain measurements, which is about 3 times less sensitive than conventional strain gauges (±1 µstrain).

Tensile test sample 2, of which the test results are summarized in Figure 9b, was tested over 20 times in order to investigate the usable life of the presented strain monitoring methodology using embedded capillaries. It is clear from these test results that, besides some spreading of the test results, degradation occurred even after a limited amount of tests. The Young Modulus of the material gradually increased and strain sensitivity decreased. It is concluded that the lack of repeatability and, thus, the usable life of the presented system, is a result of the use of polymer tensile test samples. It is expected that the usable life of the presented strain monitoring methodology will be improved when using metallic components.

## 6. Future Work

Although the ABS polymer tensile test samples presented in this paper allowed to prove the concept of integrated strain measurement by monitoring the fluid pressure inside integrated capillaries, their preparation is cumbersome and subsequent testing of polymers leads to varying material behavior and thus varying system performances. This can be seen from Figure 9b where the last set of tests showed an increased Young Modulus and reduced sensor sensitivity. Stereolithography (SLA) is a 3D printing technique that is based on the hardening of a liquid polymer when in contact with laser light. The technique allows the production of as-built air- and watertight components which would not require an after-treatment process and as such reduce the scatter on the obtained measurements. The geometrical accuracy of components produced by stereolithography is also much better, which would allow more detailed studies on the effect of the Poisson coefficient, initial pressure level and capillary diameter. With the final aim of introducing the presented strain monitoring technique inside metal printed components for structural health monitoring applications, it is expected that future studies will be devoted to fatigue testing of metallic samples in order to measure the loads, count fatigue cycles, detect overloads, etc. Many fatigue tests have already been performed in the field of structural health monitoring for detecting fatigue cracks with a system based on integrated capillaries [26,27,28,29,30,31] . It is a strength of the system, based on the capillary feature, that both methodologies (crack detection and load monitoring) could merge together in one system.

## 7. Conclusions

Current research is driven towards integration of strain sensors in components and structures for continuous load monitoring applications. Only recently, 3D printing production techniques have enabled integration of such functions into components. This work describes a method for embedding strain sensors in 3D printed components. A capillary was integrated into the structure and filled with a compressible or incompressible fluid. The pressure inside the integrated capillary was monitored. The elongation, or strain, of the component resulted in deformation of the integrated capillary and thus a deviating fluid pressure inside the capillary. The proposed integrated strain sensor was embedded inside a 3D printed tensile test sample and compared to the state-of-the-art extensometer strain measurements. The pressure changes were linearly related to the strain in the component. The uncertainty on the strain measured by the integrated sensor based on water (incompressible fluid) was ±3.1 µstrain, which is about 3 times less sensitive than conventional strain gauges (±1 µstrain). More sensitive strain measurements could be obtained with the proposed system by post-processing or by installing a more sensitive pressure sensor. Strain measurements were 32 times less accurate when the capillary was filled with air compared to the same sensor based on a capillary filled with water (incompressible fluid).

## Figures and Tables

**Figure A1:**
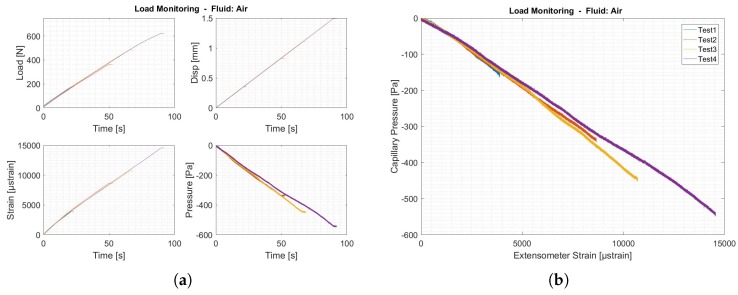
Tensile testing results: Load monitoring system with capillary filled with air is compared to extensometer strain measurements. (**a**) Raw data (**b**) Capillary pressure of the load monitoring system compared to the extensometer measurements.

**Figure A2:**
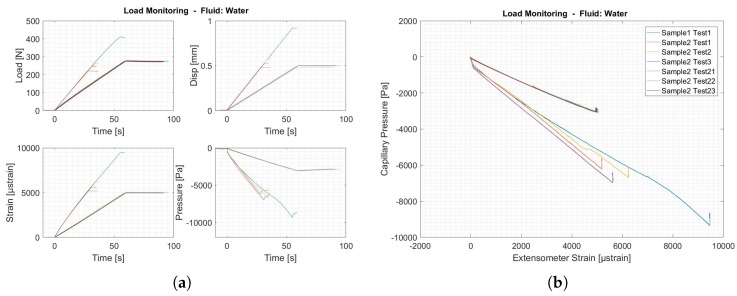
Tensile testing results: Load monitoring system with capillary filled with water is compared to extensometer strain measurements. (**a**) Raw data (**b**) Capillary pressure of the load monitoring system compared to the extensometer measurements.

## Abbreviations

The following abbreviations are used in this manuscript:

FBG	Fibre Bragg Grating
FDM	Fused Deposition Modeling
ABS	Acrylonitrile Butadiene Styrene
SLA	Stereolithography
SEM	Scanning Electron Microscope
